# Level of CYP4G19 Expression Is Associated with Pyrethroid Resistance in *Blattella germanica*

**DOI:** 10.1155/2010/517534

**Published:** 2010-04-15

**Authors:** Guang-zhou Guo, Yi-jie Geng, Da-na Huang, Cai-fang Xue, Ren-li Zhang

**Affiliations:** ^1^Department of Pathogenic Organism, Fourth Military Medical University, Xian 710032, China; ^2^Shenzhen the Fifth the People Hospital, Shenzhen, China; ^3^Shenzhen Centre for Diseases Control and Prevention, Shenzhen 518020, China

## Abstract

German cockroaches have become a large problem in the Shenzhen area because of their pesticide resistance, especially to pyrethroid. A pyrethroid called “Jia Chong Qing” to prevent pests for a long time were found to be resistant to “Jia Chong Qing” with resistance index of 3.88 measured using RT-PCR and immunohistochemistry analysis showed that both CYP4G19 mRNA and CYP4G19 protein expression levels in the wild strain were substantially higher than that of a sensitive strain. dsRNA segments derived from the target gene CYP4G19 were prepared using in vitro transcription and were microinjected into abdomens of the wild strain. Two to eight days after injection, the result showed that CYP4G19 mRNA expressions were significantly reduced in the groups injected with dsRNAs.

## 1. Introduction

The German cockroach (*Blattella germanica*) is common house pest that can spread diseases by carrying bacteria and viruses, and it can cause allergic responses in human such as asthma [1]. German cockroaches have a good adaptability to different environments and can propagate rapidly. They can become resistant to chemical pesticides easily and are one of the major obstacles for the control of urban pests. It was indicated that the mechanism of pyrethroid resistance involves the metabolic enzyme cytochrome P450 [2]. CYP450 is a hemoprotein that acts as the terminal oxidase in monooxygenase system. In vivo, it catalyzes oxidizing reaction of endogenous and exogenous material, and plays an important role in pesticide detoxification [3]. 

 RNA interference (RNAi) is a technology that can be used to down-regulate the expression of a gene by the addition of a gene-specific double-stranded RNA (dsRNA). It was originally characterized in *Caenorhabditis elegans* [4] and has since been applied to a wide variety of organisms to study gene function, including protozoa, insects, and mammals [5–7]. The molecular processes and components necessary for a functional RNAi pathway have been extensively investigated in *C. elegans* [8].

*B. germanica* has been used as a model to study nuclear receptor families participating in the 20-hydroxyecdysone (20E)-triggered genetic hierarchy [9]. *B. germanica* E75 is a member of the 20E-triggered genetic hierarchy, and RNAi experiments in vivo during the penultimate and last nymphal instars of *B. germanica* reveal that BgE75 is required for successfully completing nymphal-nymphal and nymphal-adult transitions. Injection of dsRNA into the haemocoel of nymphs and adults of the cockroach *B. germanica* was used to silence gene function in vivo and to halt the expression of the adult-specific vitellogenin gene. The same technique was used to silence the expression of the *B. germanica* RXR-homologue ultraspiracle (USP) gene in vivo during the last nymphal instar. The results raise the possibility that developmental genes can be functionally analyzed via systemic RNAi in this species [10].

 Studies of the role that insect P450 played in pesticide resistance may provide important information for developing new pesticides, for monitoring pesticide resistance, and for rational application of pesticides. However, research on the mechanisms of pesticide resistance involving P450 in German cockroaches is sparse. There are only seven German cockroach P450 genes deposited at NCBI, of which the biologic characters remained to be illuminated. German cockroach CYP4G19 is a new member of the P450 gene family that has a different expression pattern between pyrethroid sensitive strains and resistant ones. It was discovered by Pridgeon et al. using the method of selected subtractive hybridization (SSH) in 2003 [11]. Additional research was done to further study the biologic characteristics of CYP4G19 and the pyrethroid molecular resistant mechanism of CYP4G19 [12]. 

## 2. Materials and Methods

### 2.1. German Cockroaches

The sensitive strain GC209 of *B. germanica* was obtained from Guangdong Centre for Diseases Control and Prevention, and the wild strain was collected in a farmers' markets in Shenzhen.

### 2.2. Determination of the Pesticide Resistance in *B. germanica*

An 8% “Jia Chong Qing” stock solution was diluted with acetone to 0.005% working solution in a 500 mL wide-mouthed bottle according to the method of sensitive layering [13]. A 2.5 mL working solution was added into a bottle, and the bottle was rotated slowly until the liquid distributed uniformly on the inner wall. The bottle is ready for use after drying. Liquid paraffin was placed in the bottleneck to prevent cockroaches from escaping. Ten male adults were selected into the bottle and the number of knock-downs was recorded every few minutes. The experiment was repeated 3 times. The half killing time (KT_50_) and 95% CI were recorded and calculated. Resistance index (R) = wild strains KT_50_/sensitive strain KT_50_. The tests were performed at (25 ± 1)°C with (60 ± 5)% relative humidity. Acetone was used as blank control.

## 3. Detection of Differential Expression of CYP4G19 mRNA between the Sensitive and Wild Strain Using RT-PCR

Total RNA from *B. germanica* was isolated using PUREscript reagents (Gibco) according to the manufacturer's instructions. RNA samples were stored at −80°C until use. First-strand DNA synthesis was carried out with 1 *μ*g RNA in a 20 *μ*L reaction solution using RNA-PCR kit (Perkin Elmer, Branchburg, CA). Primer sequences of CYP4G19 were used and reaction conditions for PCR were 12.5 *μ*L 2× Taq PCR master mix, 1 *μ*L primer (10 *μ*mol/L), 0.5 *μ*L cDNA template, and 10 *μ*L deionized water. PCR cycling: 30 s, for 35 cycles. The CYP4G19 primers were sense 5′-acattttacatcttcgccactcc-3′ and antisense 5′-tctccttcttgttcttgatgacctt-3′. These primers amplify 423 bp fragment from CYP4G19 mRNA. The gene encoding *β*-actin was used as a control, and primers were sense 5′-atggaatcatcaccaactgg-3′ and antisense 5′-ccttgatgtcacgaacgatt-3′. RT-PCR products were separated on 1% agarose gels. The gels were scanned using a UVI gel scanning system and relevant software to estimate band intensity and mRNA levels.

## 4. Recombinant CYP4G19 (rCYP4G19) Proteins and Preparation of Anti-rCYP4G19 Antibodies

cDNA encoding amino acid residues from 1 to 273 of the *B. germanica* CYP4G19 were amplified using reverse transcription (RT)-PCR. The product was digested with Nhe I and EcoR I and ligated into the pET-28a (Pharmacis, Uppsala, Sweden). The vector was transfected into *Escherichia coli* BL21 cells (Pharmacis), and rCYP4G19 protein was induced by addition of IPTG. Purified rCYP4G19 protein was confirmed on SDS-PAGE and used to immunize BALB/c mice. The presence of anti-rCYP4G19 was confirmed using Western blot

## 5. Detection of CYP4G19 Protein Expression between the Sensitive and Wild Strain Using Immunohistochemistry

### 5.1. Tissue Section of *B. germanica*

The German cockroach was placed at −20°C for 10 minutes before the wings and feet were cut quickly. The bodies were sheared for 2-3 sections and were placed at 4°C overnight. After fixation and dehydration, the tissues were cut into 6 *μ*m sections

### 5.2. Immunohistochemistry (IHC)

After deparaffinization, the sections were treated with 0.5% hydrogen peroxide in methanol for 30 minutes and incubated in 5% skim milk in phosphate-buffered saline (PBS) for 1 hour. The sections were incubated for 2 hours with polyclonal antibody (diluted 1 : 200) against CYP4G19 recombinant protein, washed three times with PBS, incubated with goat antimouse IgG HRP secondary antibodies (1 : 200) at room temperature for 1 hour, and washed three times with PBS before addition of chromogenic reagent DAB. The reaction was stopped after rinsing with distilled water. The samples were stained with hematoxylin for 1~2 minutes, rinsed with distilled water, dehydrated, made transparent, and mounted for microscope examination. 

 After the immunohistochemical analysis, we used the IPP software (image-pro plus 5.1) to analyze the optical density of the images. The specific operations were as follows: set the recorded images to inverted mode, select and measure the brown regions with positive staining signals, and calculate the average optical density (mean density = IOD/area). Calculations of the average values and standard deviations in the same experimental groups were made, and analysis of the significant levels of the mean density among the experimental group was done with Statistica.

## 6. Synthesis of CYP4G19 dsRNA

### 6.1. Synthesis of CYP4G19 dsRNA

Two segments of the CYP4G19 gene (GenBank accession number: AY176056.1) were selected for dsRNA synthesis. Template 1 was 590 bp long (including the T7 promoter sequence) that was located at 300 bp to 864 bp in the target gene. Template 2 was 696 bp (including the T7 promoter sequence) that was located between 930 bp to 1598 bp in the target gene. Specific primers were as follows: sense 5′-taatacgactcactatagggcatgccaagggatattgag-3′ and antisense 5′-taatacgactcactatagggcatggccttctcttcttgt-3′ for template 1 and sense 5′-gaattaatacgactcactatagggagagccagaagaaacgattgg-3′ and antisense 5′-gaattaatacgactcactatagggagatgaagccatcagccctct-3′ for template 2.

### 6.2. Transcription of dsRNA In Vitro

 A 20 *μ*L reaction of transcription mixture containing 1~2 *μ*g DNA template (or control templates), 2 *μ*L 10 × T7 reaction buffer, 2 *μ*L dNTP mix, 2 *μ*L T7 enzyme, and nuclease-free water (up to 20 *μ*L) was prepared on ice. The solution was mixed completely and subjected to short centrifugation. To determine the best incubation time, a 0.5 *μ*L sample of the reaction mixture was removed at 2 hours, 3 hours, and 4 hours, and the products at different times were determined using electrophoresis. Transcription at 37°C was done when the best incubation time was worked out. If the template DNA was less than 800 bp, dsRNA synthesis was done without annealing steps.

### 6.3. Purification of dsRNA

DNA and ssRNA were digested in mixtures containing 21 *μ*L nuclease-free water, 5 *μ*L 10 × digestion buffer, and 2 *μ*L DNase I mix at 37°C for 1 hour (15 minutes long for ssRNA). Digested DNA or ssRNA were purified using a Filter Cartridge (Promega) according to the manufacturer's instructions. Samples of eluted DNA and dsRNA were measured in a spectrophotometer and run on a 1% agarose gel to evaluate the integrity and purity of the dsRNA. The purified products were stored at −80°C.

## 7. RNA Interference of the CYP4G19 Gene

A group of 250 wild German cockroaches (approximately evenly divided male and female) was divided into five groups, three of which were experimental groups (experimental groups I, II, III) and the other two groups were control groups including unrelated control group (silence negative control supplied by Ambion company with sequence that had no significant similarity with any gene of *B. germanica*) and a control group with ringer's saline. The experimental group I was injected with dsRNA1 solution transcribed in vitro from the template I; experimental group II was injected with dsRNA2 solution transcribed in vitro from the template II; experimental group III was injected with a mixture of dsRNA1 and dsRNA2 (1 : 1). At the same time, groups 5 and 6 did not receive any reagents and were blank controls. The dsRNA solution was rapidly injected into the abdominal using a DEPC-treated micro-syringe containing 1 *μ*L per cockroach with final concentrations of 0.5~1 *μ*g/*μ*L.

After injection, the samples were placed in separate 500 mL wide-mouthed bottles with food and wet cotton. The bottlenecks were coated in liquid paraffin and covered with gauze to prevent cockroaches from escaping. The survival rate in each group was observed, and samples were collected 2–8 days after injection and were frozen in liquid nitrogen.

## 8. Observation of Pyrethroid-Resistance after RNAi

dsRNA was injected into the abdominal cavity of wild German Cockroaches, and untreated wild-strain was used as a control, and the experiment was repeated 3 times. The resistance indexes of the experimental group and the control group were calculated, and the resistance levels before and after RNA interference were compared to determine whether changes to the German cockroach CYP4G19 gene were associated with resistance to pyrethroid insecticides.

## 9. Results

**(1) Detection of the Pesticide Resistance of the Wild Strain German Cockroaches**

The wild strain German cockroaches had significantly higher KT_50_ (26.381 with 95% CI 23.3677~29.3893) and resistance index (3.88) to Jia Chong Qing than those of the sensitive strain (KT_50_= 6.803 with 95% CI 5.4647~8.0795 and resistance index = 0.37) as determined using the sensitive layer method.

**(2) Detection of Differences in CYP4G19 mRNA Expression between the Wild and Sensitive Strain**

**Figure 1:**
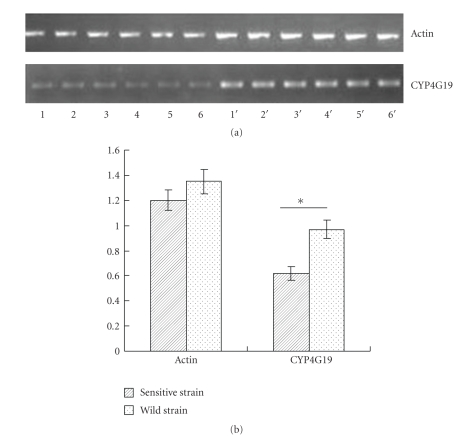
(a) Semi-quantitative RT-PCR analysis of CYP4G19 mRNA expression levels. A, Gel electrophoresis analysis of PCR products of actin and CYP4G19 genes. Lane 1~6: Pesticide sensitive strain; Lane 1′~6′: Wild strain. (b) Bar chart of semiquantitative RT-PCR analysis. The white bars were wild strain and the black bars were sensitive group. Asterisks indicated differences statistically significant *P* < .01 (*t*-test). Data of (b) represent means OD values ± SEM of three independent experiments, and each experiments including five samples of wild and sensitive strains.

The actin gene and the CYP4G19 gene from the sensitive and wild strains (each group including six samples) were amplified, and the PCR products were analyzed on 1% agarose gel electrophoresis (Figure 1(a)). The fluorescent OD value of bands was scanned, and the experiment was repeated 3 times. A paired *t*-test analysis (Figure 1(b)) showed that the gene expression of sensitive strain German cockroach was significantly lower than the wild strain (OD value of bands: sensitive stains, 0.58 ± 0.13; wild strians, 0.97 ± 0.31, *P* < .01).

**(3) The Dynamics of mRNA Expression of the CYP4G19 Gene in Wild Strain of German Cockroach after RNAi**

**Figure 2:**
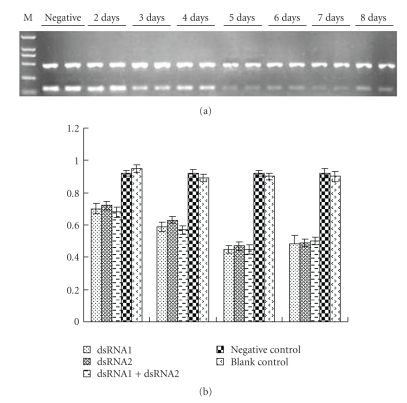
mRNA expression of the CYP4G19 gene of German cockroach after RNAi. (a) Semiquantitative RT-PCR analysis of CYP4G19 mRNA levels 2~8 days after dsRNA injection. Gel electrophoresis analysis of PCR products of actin (top band) and CYP4G19 genes (bottom band). (b) Relative mRNA levels after injection of dsRNA1, dsRNA2, dsRNA1+daRNA2, negative control dsRNA, and Ringer saline from day 2 to day 8.

Synthesized dsRNA (0.7 *μ*g each) was injected into the peritoneal of the insect and survival rates were evaluated 8 days after injection. One or two test insects died in each group, and survival rates were between 80%~90%. Six insects from each group were collected from day 2 to 8 after injection and were stored in liquid nitrogen. The mRNA levels of the CYP4G19 gene were detected using the semiquantitative RT-PCR method. The dynamics of mRNA expression of the CYP4G19 gene in the experimental groups showed that the expression of CYP4G19 gene began to decrease 2 days after injection, and continued to weaken to the lowest level at day 5 (Figure 2(a)), but there was no significant differences in the in expression of CYP4G19 gene among the dsRNA1, dsRNA2, and dsRNA1+dsRNA2 groups (Figure 2(b)).

**(4) Expression of rCYP4G19 Protein and Production of Anti- rCYP4G19 Antibodies in Mice**

**Figure 3:**
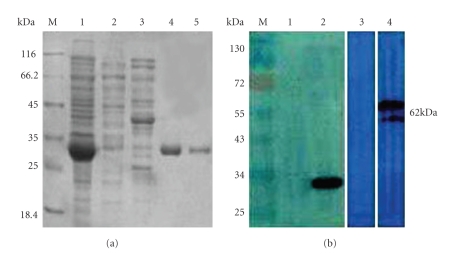
SDS-PAGE and Western blot analyses of rCYP4G19. (a) M, molecular weight markers; lane 1, cell extract from pET-28a-rCYP4G19-transformed *E.coli*; Lane 2,3, cell extract from pET-28a- transformed *E.coli*; Lane 4,5, purified rCYP4G19 protein. (b) Immunoblot analysis. IgG antibodies specific to rCYP4G19 and native CYP4G19 were detected on immunoblotting membrane using sera of immunized mice (lane 2,4). Adjuvant-treated control mouse serum (negative control) is shown in lane 1,3.

We expressed and purified a rCYP4G19 from bacteria and confirmed the protein using sodium dodecyl sulfate-poly-acrylamide gel electrophoresis (Figure 3(a)). Mice immunized with rCYP4G19 produced high level of IgG antibodies specific to the immunizing antigen. Immunoblotting analysis showed that murine anti- rCYP4G19 serum bound specifically to rCYP4G19 and native CYP4G19 protein (Figure 3(b)). The specific antisera of rCYP4G19 was used to immunohistochemistry.

**(5) Differential Expression of CYP4G19 Protein In Vivo after RNAi in Wild Strains Detected Using Immunohistochemistry**

**Figure 4:**
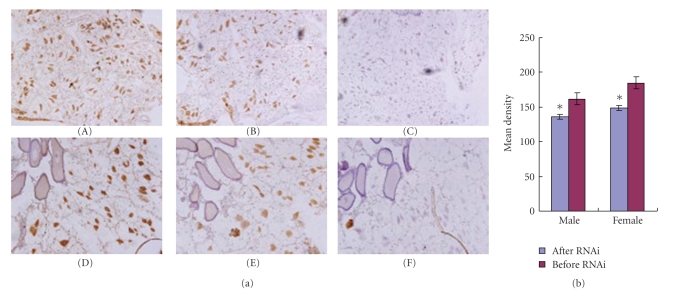
(a) Expression of CYP4G19 protein before and after RNAi in tissue of wild strains. (A)–(C) were expression of CYP4G19 protein at the microsome of male German cockroach and (D)-(F) were the microsome of female german cockroach. (A), (D) were expression of CYP4G19 protein before RNAi in wild strain and (B), (E) were expression after RNAi at the fifth day, (C), (F) were negative control. (B) The mean density values CYP4G19 protein were analyzed and counted using IPP software in tissue of wild German cockroach before and after RNAi. Bar 1, before RNAi; Bar 2, after RNAi. Asterisks indicate differences statistically significant at *P* < .01, *n* = 10 (Student's test).

CYP4G19 protein before and after RNAi with dsRNA1 and dsRNA2 was detected using immunohistochemistry. Expression of CYP4G19 protein was significantly stronger before RNAi than after RNAi (Figure 4(a)). Both male and female adults showed the same results at microsomes of the German cockroach. The results of immunohistochemistry were photographed using a fluorescent camera, 10 regions of expression were selected randomly from each biopsy, and the mean density values of the region were analyzed and counted using IPP software. The average value of mean density corresponding the biopsy of both the before and after RNAi in wild strains was analyzed by paired *t*-test (Figure 4(b)).

**(6) Pesticide Resistance of Wild Strain German Cockroach before and after RNAi**

dsRNA was injected into 60 randomly selected wild strain German cockroaches, and resistance detection was performed a week after injection. The resistance index was 3.076 for the insects treated with RNAi (KT_50_ = 6.8 with 95% CI 6.0~7.6), compared with untreated control that had a resistance index of 3.969 (KT_50_ = 21.0 with 95% CI 17.4~24.5). The result showed that the resistance index to “Jia Chong Qing” declined significantly (*P* < .05) after RNAi treatment, although the insects were still quite resisting compared with sensitive strains.

## 10. Discussion

 Many strains of *B. germanica* reported to be pyrethroid resistant have been collected from field worldwide. Some of Blattella germanica tested showed 2–10-fold resistance to the pyrethroids cyfluthrin, fenvalerate, cypermethrin, and lambda cyhalothrin appeared in wild strains [14, 15]. In some areas, pyrethroid insecticides may still be effective against *B. germanica* because of the low levels of resistance; however, the potential for developing serious resistance exists if pyrethroids are continued to be used. The prospects for the future chemical control of these populations must be carefully considered.

 There are no uniform monitoring methods for pesticide resistance of blattaria. Moss et al. reported a type of glue containing an insecticide and evaluated its ability to yield useful toxicological data against German cockroaches, Blattella germanica [16]. Choo et al. compared two resistance detection bioassay methods in cockroaches (topical application and World Health Organization glass jar method) [17]. Here we used the time-mortality response method to test for resistance to pyrethroid called “Jia Chong Qing” in field-collected strains (wild strains of *B. germanica* in comparison with sensitive strain) according to Cochran's methods [18]. We considered an insect sensitive if resistance index was less than 2, resistance if the index is more than ≥2, strong resistance if the index is >10. In this paper, we showed a wild strain of German cockroaches collected in a market where people have long used a pyrethroid called “Jia Chong Qing” to prevent pests was resistant to Jia Chong Qing with resistance index of 3.88. “Jia Chong Qing” is a mixture of chemical insecticides consisting of 2% tetramethrin and 6% cypermethrin, and is often used for pest control in Shenzhen area in recent years. Our result led us to consider replacing or rotating with other types of insecticides to combat German cockroaches. The resistant German cockroaches provide valuable experimental material for further study on the resistance mechanisms.

Some publications reported that changes in insecticide resistance levels correlated with mutation and expression of cockroach genes, especially detoxication enzymes, cytochromes P450, and so on [19, 20]. A new cytochrome P450 gene, CYP4G19, was recently identified and isolated as a differentially expressed gene between insecticide susceptible ACY and resistant Apyr-R German cockroach strains using PCR-selected subtractive hybridization and cDNA array techniques [11]. The cDNA sequence of CYP4G19 has an open reading frame of 1638 nucleotides encoding a putative protein of 546 amino acid residues, and its overexpression is correlated with resistance to insecticides [11]. Our results showed that CYP4G19 mRNA expression levels in the wild strain were obviously higher than that in sensitive strains (*P* < .01).

RNA interference (RNAi) is a technology to down-regulate the expression of a gene by the addition of endogenous or exogenous gene-specific double-stranded RNA (dsRNA). It was originally characterized in Caenorhabditis elegans [21], and since then been applied in a wide variety of organisms to study gene function. In contrast with the traditional method of studying gene function, RNAi is a technology that can be used to turn off or silent the target genes or to reduce gene expression. Silencing the expression of the *B. germanica* RXR in vivo during the last nymphal instar with an RNA interference (RNAi) approach has been described by Martin et al. (2006) [10], and the result showed that most of nymphs could not develop to the adult form. Currently, there is no study showing the use of RNA interference technology to study functions of cytochrome P450 of German cockroach.

Our results showed that the CYP4G19 gene mRNA expression was inhibited after injection of both the dsRNA1and dsRNA2. The dynamics of mRNA expression of the CYP4G19 gene in the experimental group showed that the expression of CYP4G19 gene began to be reduced within 2 days after injection. This result indicated that specific dsRNA plays the role of interference as time goes by, and the interference effect is strongest in the fifth to seventh day.

In this paper, the protein expression of CYP4G19 was detected by immunohistochemistry, and most of the CYP4G19 protein was expressed in microsome of *B. germanica*. CYP4G19 immunohistochemistry analysis results show that CYP4G19 protein expression levels before RNAi treatment are obviously higher than that after RNAi both in female and male adults (*P* < .01). Semiquantitative RT-PCR and immunohistochemistry analysis results preliminary indicated that both CYP4G19 mRNA expression levels and CYP4G19 protein expression levels in pyrethroid resistant strains are obviously higher than that in sensitive strain. This study indicated that the gene transcription and translation levels of CYP4G19 were up-regulated in pyrethroid resistant strains.

 The mean holf killinf time (KT_50_) and resistance index of both treated and untreated wild strains of German cockroach were calculated using the method of wide-mouthed bottle sensitive layer. The result showed that the pyrethroid resistance level of wild strains was reduced with decreasing of the gene expression level of CYP4G19; however, weakening of the resistance after RNAi is not a complete change in resistance phenotype. Cytochrome P450 is a large gene family. In addition to CYP4G19, there are other family members: CYP4C21, CYP6K1, CYP6L1, and CYP9E2. There is little published research on the functions of the other cytochrome P450 enzymes, and there may be some potential correlation between other P450 proteins and the pesticide resistance of German cockroaches. In addition, other detoxification enzyme systems may also play a role in detoxification in resistant German cockroaches, such as nonspecific esterase system, glutathione-S-transferase system, and so on [22, 23]. Our results suggest there may be other relevant factors related to pyrethroid resistance of German cockroaches. Though the resistance level of wild strain German cockroach was reduced with the declining of CYP4G19 gene mRNA expression, it is not enough to completely reverse the resistance of wild strains to pyrethroid.
